# Surgical Risk in Elderly Patients with Meningiomas in Japan

**DOI:** 10.3390/jcm13102882

**Published:** 2024-05-14

**Authors:** Fusao Ikawa, Nobuaki Michihata, Soichi Oya, Hideo Yasunaga, Nobutaka Horie

**Affiliations:** 1 Department of Neurosurgery, Graduate School of Biomedical and Health Sciences, Hiroshima University, Hiroshima 734-8551, Japan; 2 Department of Neurosurgery, Shimane Prefectural Central Hospital, Izumo 693-8555, Japan; 3 Department of Cancer Prevention Center, Chiba Cancer Center Research Institute, Chiba 260-8717, Japan; 4 Department of Neurosurgery, Gunma University Graduate School of Medicine, Maebashi 371-8511, Japan; 5 Department of Clinical Epidemiology and Health Economics, School of Public Health, The University of Tokyo, Tokyo 113-0033, Japan

**Keywords:** aged, database, meningioma, surgery, treatment outcome

## Abstract

**Background/Objective**: No guidelines indicate surgical risk factors for the elderly because of the lack of data from general neurosurgeons. To better understand the management of surgical risk in elderly patients with meningiomas based on a national database in Japan. **Methods**: Results of surgically treated meningiomas were explored in 8138 patients registered in the Diagnosis Procedure Combination database in Japan during 2010–2015. Age (<65, 65–74, and ≥75 years), sex, Barthel index (BI), medical history, tumor location, oral medication prescriptions on admission, and stroke complications were evaluated. Multivariate logistic regression analysis identified risk factors for stroke complications, BI deterioration between admission and discharge, and in-hospital mortality. **Results**: Advanced age was the prominent risk factor for BI deterioration (odds ratio: 3.26; 95% confidence interval: 2.69–3.95) but not for in-hospital mortality. Lower BI (60–80) on admission increased the risk of BI deterioration in all age groups; however, BI < 60 demonstrated a significant inverse risk (0.47; 0.32–0.69) in the elderly (≥75 years). Location (falx, parasagittal, and deep) and anticoagulants were not significant risk factors for BI deterioration in patients aged ≥ 75 years, despite being significant risk factors in patients aged <65 and/or 65–74 years. **Conclusions**: Although advanced age could lead to postoperative functional decline at discharge, it was not sufficiently significant enough to be associated with in-hospital mortality. Because of the possibility of recovery even in elderly patients with severe disabilities, appropriate surgical selection and optimal management may lead to favorable functional outcomes in elderly patients with meningiomas.

## 1. Introduction

Recent increases in life expectancy and the frequency of the use of diagnostic neuroimaging have resulted in a worldwide increase in the incidental detection of meningiomas in the elderly. The Japanese population has a high proportion of elderly people and the highest life expectancy at birth in the world [1]. Therefore, it is a suitable country for conducting research on various diseases among elderly patients. Surgical decision-making strategies for elderly patients should be carefully considered based on their vulnerability. Regarding frailty concerns, our group previously evaluated the outcome and risk factors for elderly patients with meningiomas [2].

Alternatively, chronological age is considered one of the most effective surgical indications for elderly patients with meningiomas worldwide, and typical surgical outcomes based on chronological age must be understood. Medical complication and mortality rates in high volume centers have been reported to be 6.8% and 0.48%, respectively [3]. Outcome studies from administrative databases have reported new neurological deficits and postoperative 30-day mortality rates of 14.8% and 1.5%, respectively [4]. Our recent systematic review reported that the 1-year postoperative mortality and rate of neurological complications in elderly patients with meningiomas ranged from 0 to 16.7% and 2.7 to 49.4%, respectively [5]. Although several studies have reported on surgeries in elderly patients with meningiomas, preoperative and postoperative activities of daily living (ADL) have been rarely investigated [6]. Additionally, no guidelines indicate surgical risk factors for this population because of the lack of data accumulated from general neurosurgeons.

As we have previously published the results of a nationwide database regarding cerebral aneurysm [7] and thus understand the limitations of large databases, we ensured data integrity [8,9]. The Barthel index (BI) as an indicator of ADL and the location of meningiomas were evaluated as additional factors for further integrity in data. We clarified surgical risk factors for elderly patients with meningiomas from the Diagnosis Procedure Combination (DPC) database.

## 2. Methods

### 2.1. Protocol Approval, Patient Consent, and Data Availability

The present study was approved by the local institutional review boards of Hiroshima University (no. E-631) and Tokyo Medical and Dental University (no. 3501-[1]).788) (ID: UMIN000038486; No. R000043856; URL, https://www.umin.ac.jp/ctr/index.htm, accessed on 1 April 2024) and conducted in accordance with The Code of Ethics of the World Medical Association (Declaration of Helsinki) for experiments involving humans. Because of the anonymous nature of the data, informed consent was not required. The anonymized data for this study could be shared on request from any qualified investigator to the corresponding author. Only the results of primary data from the DPC database can be made available upon reasonable requests in accordance with the review board of Hiroshima University and Tokyo Medical and Dental University.

### 2.2. Data Source and Patient and Meningioma Selection

The Japanese DPC is a registry-based national database that includes administrative claims and abstract discharge data on inpatients in Japan. It has been described thoroughly elsewhere [7,10]. The database incorporates the following variables: unique hospital identifier; patient postal code; patient age and sex; diagnoses and comorbidities on admission; past medical history; medications prescribed both at admission and for any post-operative complications, coded as per the International Classification of Diseases and Related Health Problems, 10th Revision (ICD-10); and BI at admission and at discharge. All academic hospitals are required to participate in the database, but participation by nonacademic community hospitals is voluntary. A previous study using this specific inpatient database previously established its validity [11]. We observed that diagnosis specificity exceeded 96%, whereas sensitivity was 50–80%. Both the specificity and sensitivity of the procedure records exceed 90% [11].

**Figure 1:**
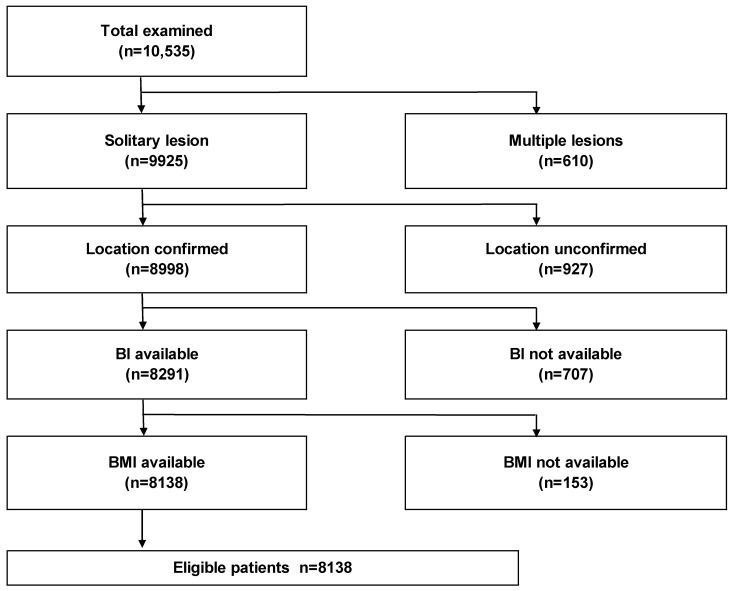
Selection process for patients with surgically treated meningiomas. Abbreviations: BI, Barthel index; BMI, body mass index.

We included patients aged 9–95 years admitted to the hospital with a primary diagnosis of intracranial meningioma between 1 July 2010 and 31 March 2015. Diagnoses of meningioma and intracranial tumor removal procedure were evaluated based on ICD-10 codes (D320 and K169, respectively). In total, 10,535 patients with meningiomas were identified. We excluded multiple meningiomas, cases with unknown location, no BI assessments, and no detection of body mass index (BMI). Consequently, 8138 patients with meningiomas were retained (Figure 1).

We collected patient data on age, sex, BMI, past medical history, oral medication at admission, location of the meningioma, BI score at admission and at discharge, and post-operative complications. As part of the medical history, we included a review of hypertension (I10), diabetes mellitus (ICD-10 code: E14), cerebral infarction (I639), angina pectoris (I209), and chronic heart disease (I509). We reviewed the use of antiplatelet drugs (aspirin, cilostazol, ticlopidine hydrochloride, clopidogrel sulfate, clopidogrel–aspirin combination, and prasugrel hydrochloride), anticoagulation agents (warfarin, edoxaban tosilate hydrate, dabigatran etexilate, rivaroxaban, and apixaban), and statins (atorvastatin calcium hydrate, rosuvastatin calcium, and pitavastatin calcium hydrate). In addition, we reviewed patients for the presence of complications, including intracerebral hemorrhage (ICH; ICD-10 code: I619), subarachnoid hemorrhage (SAH; I609), cerebral infarction, chronic heart disease, and pneumonia (J189).

BI evaluates 10 ADLs across two to four stages. It is a widely accepted tool that has been used to assess functional impairments associated with a range of neurological disorders and brain tumors [12,13]. BI was classified in three categories: 0–55, 60–80, and 85–100 points according to ADL, with higher scores indicating a higher level of independent functioning. All cases of stroke were caused by ICH, SAH, or cerebral infarction. BI deterioration indicated patients who had a lower BI score at discharge compared to admission, and in-hospital mortality indicated death from any cause.

The hospital data that we reviewed primarily assessed case volume and type of hospital (academic or non-academic). The definition of hospital volume included the number of patients with meningiomas treated surgically at an individual facility during the study period; this was categorized into three groups according to terciles of case volume, which facilitated roughly equal numbers of patients in each of the three groups. We classified hospital volume from 1 to 3, ordered from lowest to highest case volume. Anatomical location of the meningioma was classified as convexity, falx, parasagittal, lateral (sphenoidal ridge or middle fossa), midline (tuberculum sellae or olfactory groove), posterior fossa (foramen magnum, petrous, petroclival, or petrotentorial, tentorial), and deep (ventricle, anterior clinoid, posterior clinoid, cavernous, orbital, or falcotentorial). Patients were categorized into three age groups, following the classification of the World Health Organization and the Japan Geriatrics Society, as follows: <65 years (non-elderly), 65–74 years (pre-elderly), and ≥75 years (elderly) [14]. Finally, we categorized all patients according to their BMI into the following groups: <18.50, 18.50–24.99 (healthy weight), 25.00–29.99, and ≥30.00.

### 2.3. Statistical Analyses

All statistical analyses were conducted using Stata software (version 15; StataCorp, College Station, TX, USA). To compare categorical variables, we performed a Chi-square or Fisher’s exact test. In contrast, to compare continuous variables, we performed a t-test or Mann–Whitney U test. Multivariate logistic regression analyses were performed on the overall cohort to analyze and compare all stroke complications, the deterioration in BI between admission and discharge, and in-hospital mortality; the same analyses were performed separately in the three categories of age groups, except for in-hospital mortality where the numbers of participants in each subgroup was low and thus the analysis was waived. For multivariate logistic regression analyses, independent variables were selected based on the existing literature, and no variable selection method was applied; odds ratios (ORs) and 95% confidence intervals (CIs) were calculated.

## 3. Results

During the study period, 8138 eligible patients were surgically treated. The mean (interquartile range) age was 63.0 (53.0, 71.0). Table 1 shows the baseline characteristics of patients with surgically treated meningiomas by age group. Sex, age classification, BMI classification, location of meningioma, hospital volume, academic hospital, BI classification on admission and discharge, medical history, oral medication on admission, pneumonia complication, and BI deterioration all significantly differed between age groups (*p* < 0.001), whereas there was no significant difference between age groups in BMI; complications of ICH, SAH, cerebral infarction, stroke, and chronic heart disease; and in-hospital mortality. Patients with the lowest BI scores (0–55 points) tended to decrease at discharge compared to admission in all age groups. The proportion of patients with lower (60–80 points) and lowest (<60) BI scores increased with age both at admission and at discharge. Alternatively, patients with high BI scores (85–100 points) tended to improve at discharge compared to admission in nonelderly and pre-elderly groups; however, this was less common in the elderly group. Finally, the proportion of patients with a medical history and oral medication prescriptions on admission significantly increased with age.

Table 2 depicts the results of the multivariate logistic regression analyses for all stroke complications, BI deterioration, and in-hospital mortality. In all stroke complications, location in the posterior fossa (OR: 1.56; 95% CI: 1.06–2.30), the presence of antiplatelets at admission (6.72; 4.53–9.96), and anticoagulation (2.92; 1.83–4.65) were significant risk factors, whereas lateral location (0.35; 0.17–0.74) was a significantly inverse risk factor. For BI deterioration, significant risk factors included being aged 65–74 years (1.79; 1.50–2.12) and ≥75 years (3.26; 2.69–3.95); meningioma location of parasagittal (1.88; 1.51–2.33), lateral (1.32; 1.04–1.69) and deep (2.52; 1.62–3.91); BI classification on admission from 60 to 74 (2.58; 1.99–3.34); cerebral infarction (1.67; 1.04–2.67); and anticoagulants at admission (2.26; 1.70–3.02). For in-hospital mortality, BMI over 18.5 (2.67; 1.15–6.62); tumor location of falx (5.08; 1.56–16.54), parasagittal (5.90; 1.82–19.08), midline (10.53; 3.41–32.56), posterior fossa (7.51; 2.59–21.83), and deep (9.06; 1.65–49.82); BI classification on admission of 0–55 (7.12; 3.62–13.94) and 60–80 (4.13; 1.37–12.42); history of cerebral infarction (3.68; 1.01–13.38); and antiplatelet oral medications on admission (4.20; 1.66–10.59) were significant risk factors, whereas hospital volume 3 (0.33; 0.13–0.84) was a significant inverse risk factor. Supplementary Figure S1A–C depicts the results of the multivariate logistic regression analysis for all stroke complications, BI deterioration, and in-hospital mortality as forest plots.

**Figure 2:**
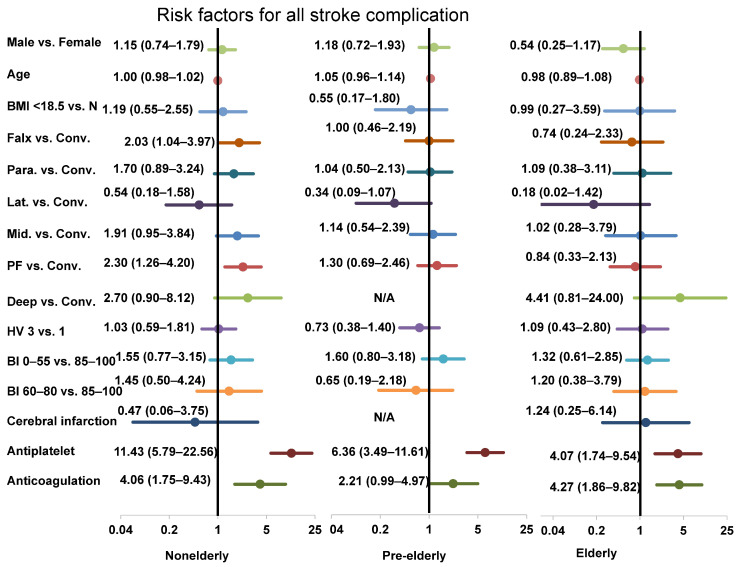
Forest plots of the risk factors for stroke complications in the nonelderly, pre-elderly, and elderly. Abbreviations: BI, Barthel index; BMI, body mass index; Conv, convexity; HV, hospital volume; Lat, lateral; Mid, midline; N, normal; Para, parasagittal; PF, posterior fossa.

Next, Supplementary Table S1 demonstrates the results of the multivariate logistic regression analyses for all stroke complications in each age group. Sex, age, BMI, location, hospital volume, academic hospital, BI classification on admission, medical history, and use of statin drugs were all insignificant risk factors in the pre-elderly and elderly groups. Antiplatelet and anticoagulation drugs were repeatedly significant risk factors in all groups. Figure 2 shows the results of the multivariate logistic regression analysis for all stroke complications in all groups as forest plots.

**Figure 3:**
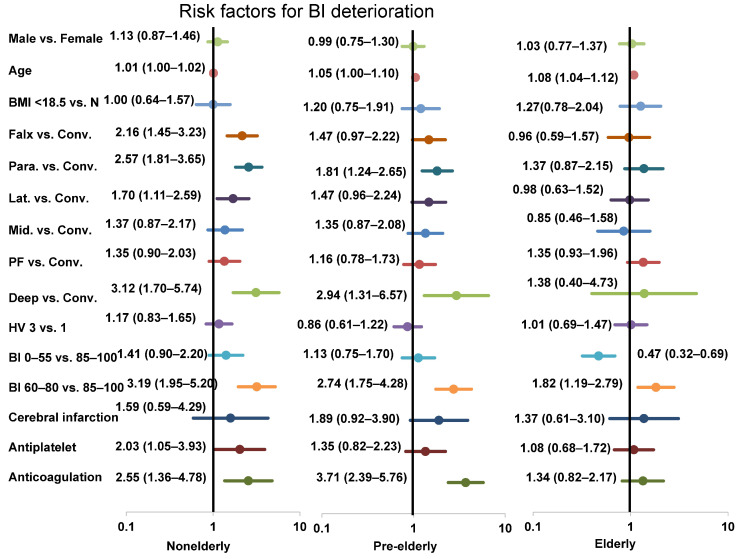
Forest plots of the risk factors for BI deterioration in the nonelderly, pre-elderly, and elderly. Abbreviations: BI, Barthel index; BMI, body mass index; Conv, convexity; HV, hospital volume; Lat, lateral; Mid, midline; N, normal; Para, parasagittal; PF, posterior fossa.

Supplementary Table S2 shows the results of multivariate logistic regression analyses for BI deterioration in each age group. In the pre-elderly and elderly groups, sex, BMI, location (except for parasagittal and deep) in the pre-elderly group, hospital volume, being in an academic hospital, medical history, taking antiplatelet drugs, taking anticoagulation drugs (except for pre-elderly group), and taking statin drugs were all nonsignificant risk factors. Age (pre-elderly [1.05; 1.00–1.10] and elderly [1.08; 1.04–1.12]) and BI classification on admission of 60–80 (pre-elderly [2.74; 1.75–4.28] and elderly [1.82; 1.19–2.79]) were significant risk factors in the pre-elderly and elderly groups. BI classification on admission of 0–55 (0.47; 0.32–0.69) in the elderly group was a significant inverse risk factor. Figure 3 depicts the results of the multivariate logistic regression analysis for BI deterioration in the nonelderly, pre-elderly, and elderly groups as forest plots.

## 4. Discussion

The median age was the highest in the present database compared with that in the other databases about meningioma surgery [3,4,15,16,17,18,19], suggesting that the present database may be an optimal database to evaluate the risk factors for elderly patients with meningiomas.

Advanced age was significantly associated with BI deterioration; however, we found no significant association between advanced age and stroke complications, and in-hospital mortality. We assessed BI deterioration between admission and discharge and found that BI deterioration increased from 7.1% to 21.1% (mean = 11.0%) as age increased, which is comparable to previous reports of 8.3–14.8% [3,4,20]. The rate of postoperative intracranial hemorrhage in need of surgical evacuation and mortality were reported as 2.1–7.1% [21,22] and 0.5–1.5% [3,4,22], respectively. Comparable to previous reports [3,5,21,22], in our present sample, the rates of all strokes and in-hospital mortality were 2.4–3.4% and 0.5–0.9%, respectively. Although meningioma surgery is associated with a higher risk for postoperative hematoma in the elderly [21], we could not find any significant correlations by age group to corroborate this, nor stroke complications. According to a recent systematic review, in-hospital mortality, the deterioration rate of postoperative performance status, and brain and general complications were not associated with advanced age [5]; however, we found pneumonia and BI deterioration to be significantly correlated with advanced age. The most common complications after surgery for meningioma were new focal neurological deficits and pneumonia [3,4]. Notably, these two complications are considered common and inevitable in elderly patients with meningioma [3].

Several identified risk factors exist for mortality in meningioma surgery in the elderly, such as location [23], preoperative Karnofsky performance status [24], BI [25], other grading systems [26], and advanced age [27]. In the present study, we found advanced age to be a risk factor only for BI deterioration, not for in-hospital mortality. Lower BI (60–80) was a risk for BI deterioration and in-hospital mortality; however, the lowest BI (<60) demonstrated an inverse risk for BI deterioration in elderly patients (Figure 2, Supplementary Table S1). We speculate that this is because the preoperative BI in this group was poor enough and therefore less likely to worsen postoperatively. However, the rate of 23.5% of the lowest BI scores on admission in the elderly patients decreased to 20.9% at discharge, indicating that even elderly patients with the lowest BI scores may improve with appropriate management. Although parasagittal and deep locations and the use of anticoagulants were significant risk factors for BI deterioration in both non-elderly and pre-elderly groups, they were no longer the risk factors in the elderly group (Figure 3, Supplementary Table S2). This phenomenon may be attributed to the delicate treatment management and positive postoperative immediate rehabilitation for the elderly. Another possible reason may be the appropriate selection of surgery for elderly patients with meningiomas. Therefore, positive management with utmost care for the elderly patients might be recommended.

Antiplatelet and anticoagulation drugs were risk factors for worse outcome in previous neurosurgical studies [28]; however, we found that antiplatelet drugs were not a significant risk factor for BI deterioration in the pre-elderly and elderly, nor were the anticoagulation drugs in the elderly (Figure 3, Supplementary Table S2). Antithrombotic drugs are beneficial for cardiac and cerebral vascular events, necessitating the correct use of these drugs in the elderly. As several studies have reported that antithrombotic treatment did not increase the risk of mortality in chronic subdural hematoma [29], antithrombotic drugs might not pose a significant risk in meningioma surgery for the elderly. Proper anticoagulant use is important, especially in the elderly, because anticoagulants are more frequently administered to the elderly as needed.

This study has some limitations. First, the DPC database does not include post-discharge data, meningioma size, and pathological findings. Therefore, we could not assess long-term patient status of recurrence or BI after discharge. These variables are usually not recorded in administrative databases. Second, this was a registry-based study, not a randomized controlled study. Therefore, we could not completely exclude any bias. However, we did improve the integrity by location of meningioma and preoperative and postoperative BI. Third, our results might not be generalizable to other countries with different medical resources and systems, and must be interpreted with caution, especially because Japan has the highest proportion of elderly people in the world.

## 5. Conclusions

Although advanced age could lead to postoperative functional decline at discharge, it was not sufficiently significant enough to be associated with in-hospital mortality. Because of the possibility of recovery even in elderly patients with severe disabilities, appropriate surgical selection and optimal management may lead to favorable functional outcomes in elderly patients with meningiomas. Further prospective randomized trials are needed.

## Figures and Tables

**Table 1:** Baseline Characteristics of Patients with Surgically Treated Meningioma Based on Age Groups.

Variable	Nonelderly (<65)	Pre-Elderly (65–74)	Elderly (≥75)	*p*–Value
	(n = 4378)	(n = 2421)	(n = 1339)	
Sex (male)	1305 (29.8%)	760 (31.4%)	479 (35.8%)	<0.001 *
Age, median (IQR)	54.0 (46.0, 60.0)	69.0 (67.0, 72.0)	78.0 (76.0, 81.0)	<0.001 *
BMI, median (IQR)	22.7 (20.4, 25.4)	23.0 (20.8, 25.3)	22.9 (20.8, 25.4)	0.082
BMI classification				<0.001 *
<18.5	351 (8.0%)	167 (6.9%)	116 (8.7%)	
18.5–24.9	2821 (64.4%)	1578 (65.2%)	851 (63.6%)	
25–29.9	942 (21.5%)	574 (23.7%)	321 (24.0%)	
30≤	264 (6.0%)	102 (4.2%)	51 (3.8%)	
Location				<0.001 *
Convexity	1414 (32.3%)	809 (33.4%)	565 (42.2%)	
Falx	469 (10.7%)	291 (12.0%)	136 (10.2%)	
Parasagittal	741 (16.9%)	351 (14.5%)	141 (10.5%)	
Lateral	491 (11.2%)	277 (11.4%)	166 (12.4%)	
Midline	473 (10.8%)	274 (11.3%)	84 (6.3%)	
Posterior fossa	672 (15.3%)	381 (15.7%)	233 (17.4%)	
Deep	118 (2.7%)	38 (1.6%)	14 (1.0%)	
Hospital volume				<0.001 *
1	1296 (29.6%)	866 (35.8%)	516 (38.5%)	
2	1402 (32.0%)	765 (31.6%)	449 (33.5%)	
3	1680 (38.4%)	790 (32.6%)	374 (27.9%)	
Academic	1838 (42.0%)	861 (35.6%)	422 (31.5%)	<0.001 *
BI on admission				<0.001 *
0–55	264 (6.0%)	234 (9.7%)	314 (23.5%)	
60–80	119 (2.7%)	117 (4.8%)	113 (8.4%)	
85–100	3995 (91.3%)	2070 (85.5%)	912 (68.1%)	
BI at discharge				<0.001 *
0–55	141 (3.2%)	194 (8.1%)	278 (20.9%)	
60–80	137 (3.1%)	126 (5.2%)	161 (12.1%)	
85–100	4079 (93.6%)	2089 (86.7%)	888 (66.9%)	
Medical history				
Diabetes mellitus	387 (8.8%)	395 (16.3%)	218 (16.3%)	<0.001 *
Hypertension	736 (16.8%)	789 (32.6%)	494 (36.9%)	<0.001 *
Cerebral infarction	35 (0.8%)	48 (2.0%)	34 (2.5%)	<0.001 *
Angina pectoris	51 (1.2%)	69 (2.9%)	68 (5.1%)	<0.001 *
Chronic heart disease	20 (0.5%)	26 (1.1%)	36 (2.7%)	<0.001 *
Oral medication on admission				
Antiplatelet	84 (1.9%)	139 (5.7%)	126 (9.4%)	<0.001 *
Anticoagulation	72 (1.6%)	105 (4.3%)	107 (8.0%)	<0.001 *
Statin	253 (5.8%)	279 (11.5%)	178 (13.3%)	<0.001 *
Complication				
ICH	16 (0.4%)	14 (0.6%)	9 (0.7%)	0.260
SAH	3 (0.1%)	1 (<1%)	0 (0.0%)	0.600
Cerebral infarction	85 (1.9%)	68 (2.8%)	30 (2.2%)	0.069
All stroke	104 (2.4%)	83 (3.4%)	39 (2.9%)	0.054
Chronic heart disease	15 (0.3%)	13 (0.5%)	8 (0.6%)	0.330
Pneumonia	81 (1.9%)	73 (3.0%)	56 (4.2%)	<0.001 *
BI deterioration	311 (7.1%)	305 (12.6%)	283 (21.1%)	<0.001 *
In–hospital mortality	22 (0.5%)	12 (0.5%)	12 (0.9%)	0.210

Abbreviations: BI, Barthel index; BMI, body mass index; ICH, Intracerebral hemorrhage; IQR, interquartile range; SAH, subarachnoid hemorrhage. * *p* < 0.05.

**Table 2:** Multivariate Logistic Regression Analyses for All Stroke Complications, BI Deterioration, and In-Hospital Mortality in the Total Sample.

Variable	All Stroke Complications		BI Deterioration		In-Hospital Mortality	
	OR (95% CI)	*p*	OR (95% CI)	*p*	OR (95% CI)	*p*
Sex (male)	1.00 (0.74–1.34)	0.999	1.05 (0.90–1.22)	0.562	1.58 (0.85–2.94)	0.148
Age group						
Nonelderly	reference		reference		reference	
Pre-elderly	1.19 (0.87–1.62)	0.287	1.79 (1.50–2.12)	<0.001 *	0.68 (0.32–1.45)	0.317
Elderly	0.86 (0.57–1.30)	0.472	3.26 (2.69–3.95)	<0.001 *	0.88 (0.38–1.99)	0.751
BMI classification						
<18.5	0.88 (0.50–1.56)	0.665	1.16 (0.89–1.51)	0.260	2.67 (1.15–6.22)	0.023 *
18.5–24.9	reference		reference		reference	
25–29.9	1.18 (0.86–1.64)	0.309	1.03 (0.87–1.23)	0.732	1.48 (0.71–3.10)	0.300
30≤	1.35 (0.78–2.34)	0.280	0.86 (0.61–1.22)	0.409	2.04 (0.66–6.35)	0.217
Location						
Convexity	reference		reference		reference	
Falx	1.28 (0.81–2.01)	0.288	1.44 (1.13–1.84)	0.003 *	5.08 (1.56–16.54)	0.007 *
Parasagittal	1.26 (0.82–1.93)	0.293	1.88 (1.51–2.33)	<0.001 *	5.90 (1.82–19.08)	0.003 *
Lateral	0.35 (0.17–0.74)	0.006 *	1.32 (1.04–1.69)	0.024 *	0.65 (0.07–5.70)	0.700
Midline	1.39 (0.88–2.21)	0.160	1.18 (0.90–1.55)	0.238	10.53 (3.41–32.56)	<0.001 *
Posterior fossa	1.56 (1.06–2.30)	0.023 *	1.23 (0.98–1.54)	0.070	7.51 (2.59–21.83)	<0.001 *
Deep	1.69 (0.71–4.01)	0.232	2.52 (1.62–3.91)	<0.001 *	9.06 (1.65–49.82)	0.011 *
Hospital volume						
1	reference		reference		reference	
2	0.85 (0.61–1.20)	0.369	0.96 (0.80–1.15)	0.681	0.55 (0.27–1.11)	0.094
3	0.93 (0.64–1.37)	0.730	0.99 (0.81–1.21)	0.936	0.33 (0.13–0.84)	0.020 *
Academic	0.87(0.63–1.22)	0.427	1.05 (0.89–1.25)	0.560	0.96 (0.45–2.04)	0.911
BI classification						
85–100	reference		reference		reference	
0–55	1.49 (0.99–2.25)	0.054	0.87 (0.69–1.10)	0.259	7.12 (3.64–13.94)	<0.001 *
60–80	1.15 (0.61–2.18)	0.672	2.58 (1.99–3.34)	<0.001 *	4.13 (1.37–12.42)	0.012 *
Medical history						
Diabetes mellitus	1.03 (0.70–1.53)	0.876	1.17 (0.95–1.43)	0.143	1.67 (0.78–3.59)	0.187
Hypertension	1.06 (0.77–1.45)	0.727	0.95 (0.80–1.12)	0.505	0.61 (0.28–1.31)	0.206
Cerebral infarction	0.36 (0.11–1.18)	0.090	1.67 (1.04–2.67)	0.034 *	3.68 (1.01–13.38)	0.048 *
Angina pectoris	0.51 (0.21–1.24)	0.138	0.99 (0.65–1.51)	0.961	0.78 (0.10–6.25)	0.817
Chronic heart disease	0.41 (0.09–1.78)	0.234	1.01 (0.55–1.85)	0.987	3.37 (0.72–15.78)	0.122
Oral medication on admission						
Antiplatelet	6.72 (4.53–9.96)	<0.001 *	1.32 (0.98–1.79)	0.069	4.20 (1.66–10.59)	0.002 *
Anticoagulation	2.92 (1.83–4.65)	<0.001 *	2.26 (1.70–3.02)	<0.001 *	1.49 (0.50–4.47)	0.477
Statin	0.90 (0.58–1.41)	0.649	1.19 (0.95–1.51)	0.135	1.00	

Abbreviations: BI, Barthel index; BMI, body mass index; CI, confidence interval; OR, odds ratio. * *p* < 0.05.

## Data Availability

The anonymized data for this study could be shared on request of any qualified investigator to the corresponding author. Only the results of primary data from the DPC could be made available for reasonable requests in accordance with the review board.

## Supplementary Materials

The following supporting information can be downloaded at: https://www.mdpi.com/article/10.3390/jcm13102882/s1, Supplementary Figure S1: Forest plots for risk factors. (A) Stroke complications. (B) BI deterioration. (C) In-hospital mortality. Abbreviations: BI, Barthel index; BMI, body mass index; Conv, convexity; E, elderly; HV, hospital volume; Lat, lateral; Mid, midline; N, normal; Para, parasagittal; PE, pre-elderly; PF, posterior fossa; Supplementary Table S1: Multivariate logistic regression analyses for all stroke complications based on age groups.; Supplementary Table S2: Multivariate logistic regression analyses for BI deterioration based on age groups.
