# Supplementary material for: Surgical Risk in Elderly Patients with Meningiomas in Japan
Source: J Clin Med. 2024 May 14;13(10):2882. doi: 10.3390/jcm13102882 (PMC11122238; doi:10.3390/jcm13102882)
Supplement: Supplementary file 1 [file jcm-13-02882-s001.zip › Figure S1.pdf]

Supplementary Figure S1A.

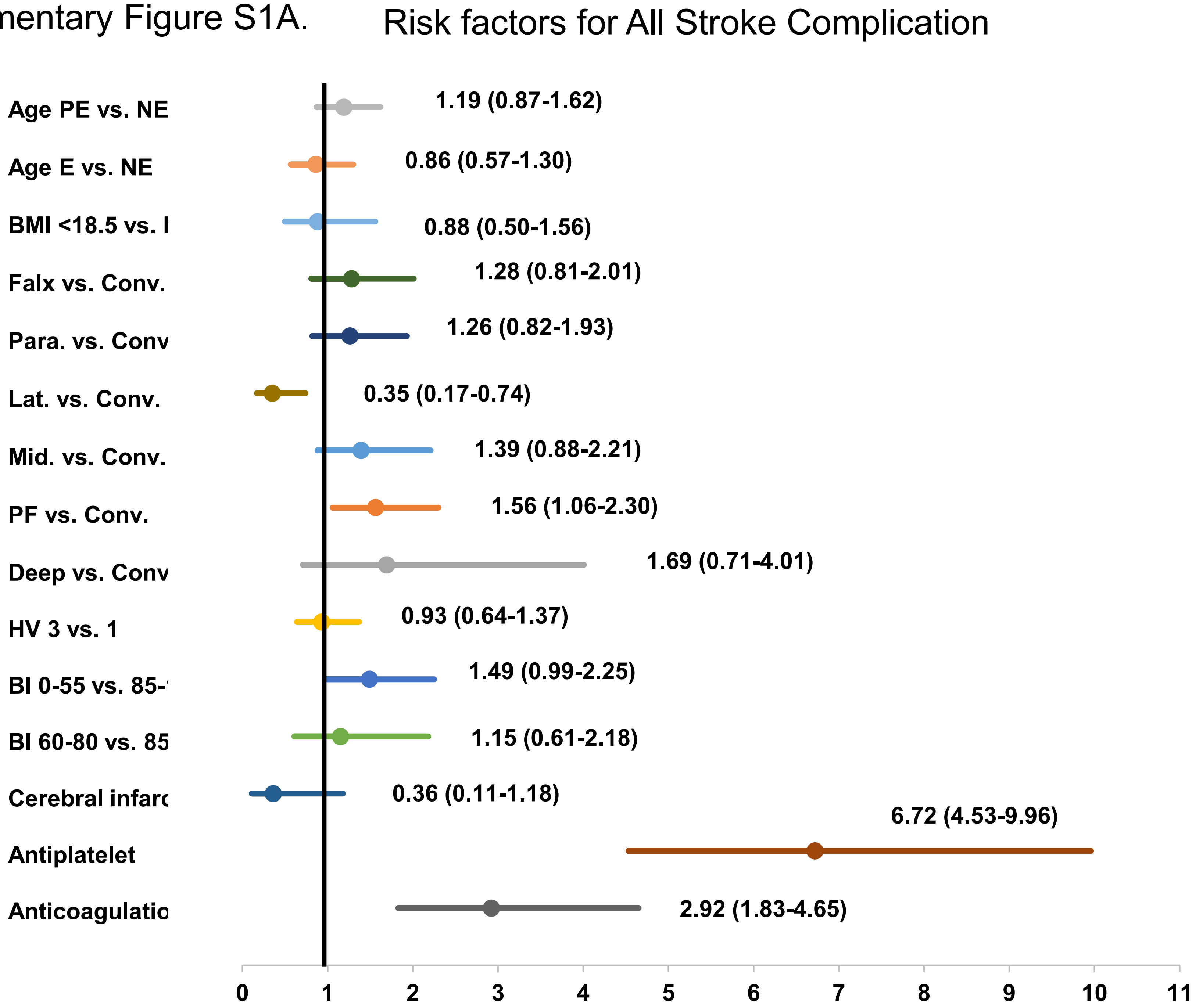

Forest plots of the risk factors for All Stroke Complication

Supplementary Figure S1B.

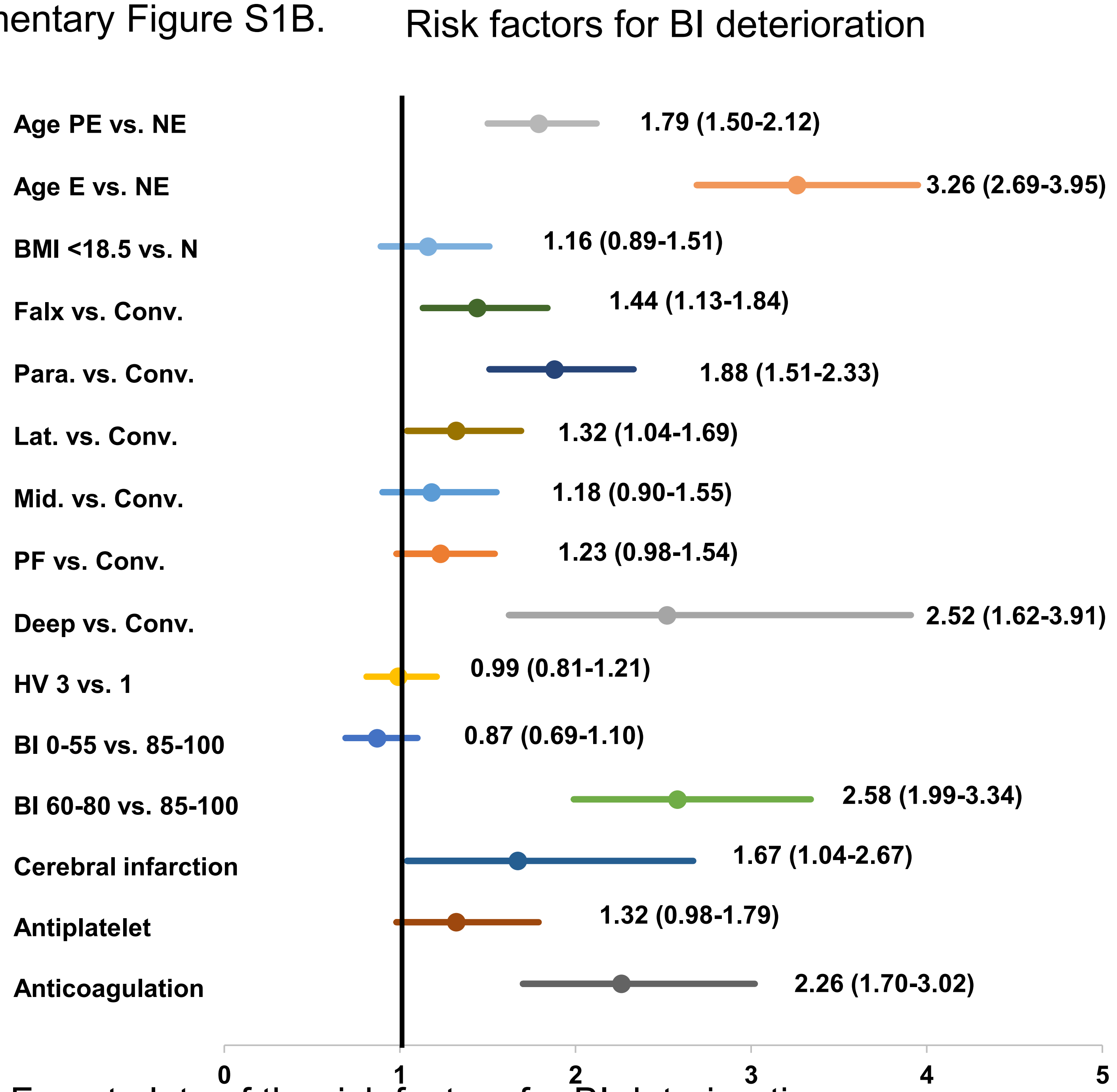

Forest plots of the risk factors for BI deterioration

Supplementary Figure S1C.

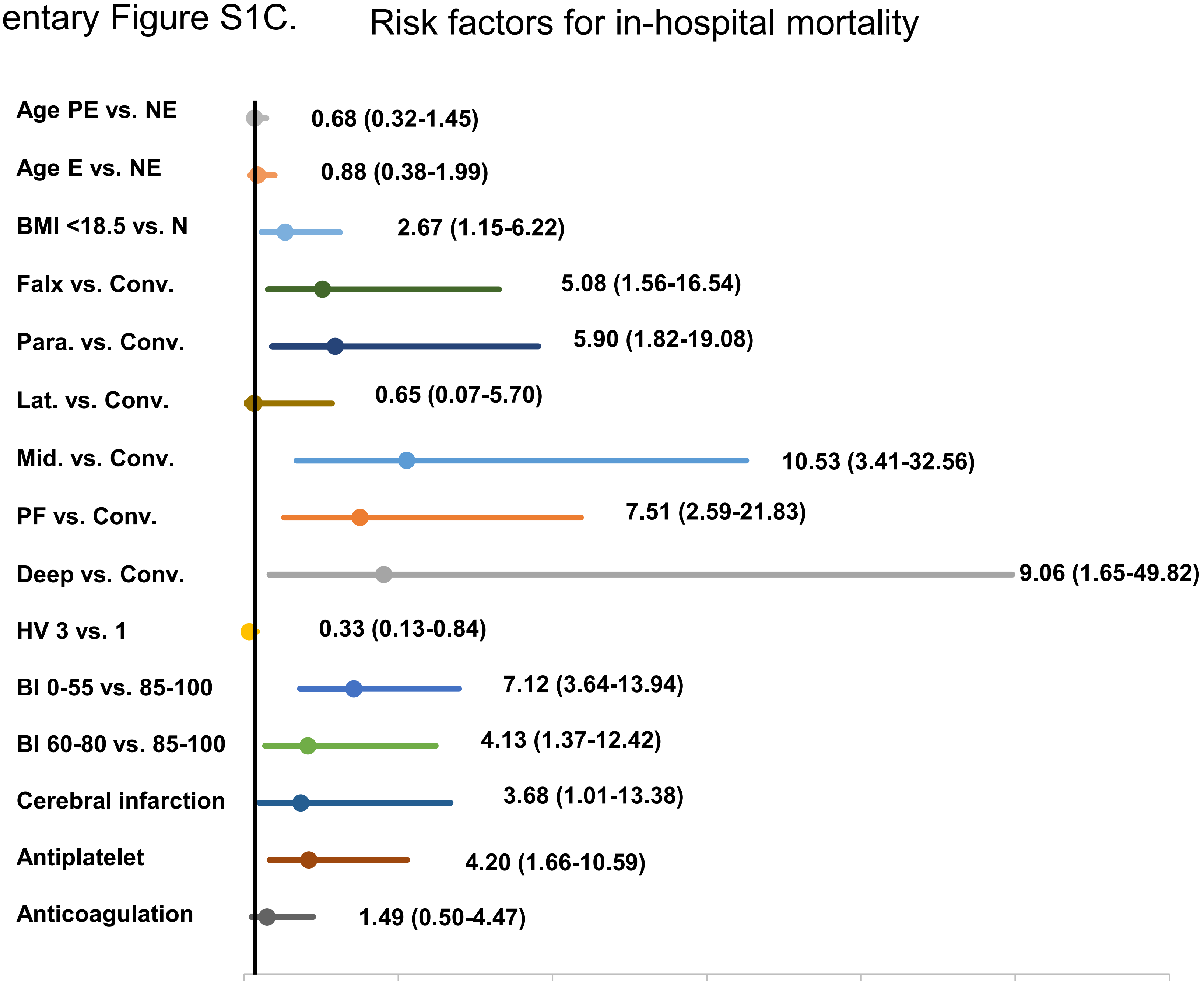

Forest plots of the risk factors for in-hospital mortality
