# Supplementary material for: Surgical Risk in Elderly Patients with Meningiomas in Japan
Source: J Clin Med. 2024 May 14;13(10):2882. doi: 10.3390/jcm13102882 (PMC11122238; doi:10.3390/jcm13102882)
Supplement: Supplementary file 1 [file jcm-13-02882-s001.zip › Table S1.pdf]

Supplementary Table S1. Multivariate logistic regression analyses for all stroke complications based on age groups.

Variable	All stroke complication					
	Nonelderly: < 65 (n=4358)		Pre-elderly: 65–74 (n=2335)		Elderly: ≥75 (n=1303)	
	OR (95% CI)	p	OR (95% CI)	p	OR (95% CI)	p
Sex male	1.15 (0.74–1.79)	0.524	1.18 (0.72–1.93)	0.522	0.54 (0.25–1.17)	0.118
Age (year)	1.00 (0.98–1.02)	0.847	1.05 (0.96–1.14)	0.274	0.98 (0.89–1.08)	0.696
BMI classification						
<18.5	1.19 (0.55–2.55)	0.658	0.55 (0.17–1.80)	0.321	0.99 (0.27–3.59)	0.988
18.5–24.9	reference		reference		reference	
25–29.9	1.35 (0.83–2.19)	0.223	0.93 (0.53–1.62)	0.794	1.31 (0.61–2.81)	0.481
30≤	1.28 (0.59–2.77)	0.526	1.62 (0.61–4.32)	0.334	1.03 (0.22–4.89)	0.966
Location						
Convexity	reference		reference		reference	
Falx	2.03 (1.04–3.97)	0.039*	1.00 (0.46–2.19)	0.999	0.74 (0.24–2.33)	0.607
Parasagittal	1.70 (0.89–3.24)	0.107	1.04 (0.50–2.13)	0.924	1.09 (0.38–3.11)	0.870
Lateral	0.54 (0.18–1.58)	0.261	0.32 (0.09–1.07)	0.064	0.18 (0.02–1.42)	0.104
Midline	1.91 (0.95–3.84)	0.070	1.14 (0.54–2.39)	0.739	1.02 (0.28–3.79)	0.975
Posterior fossa	2.30 (1.26–4.20)	0.007*	1.30 (0.69–2.46)	0.416	0.84 (0.33–2.13)	0.714
Deep	2.70 (0.90–8.12)	0.077	N/A		4.41 (0.81–24.00)	0.086
Hospital volume						
1	reference		reference		reference	
2	0.66 (0.39–1.11)	0.120	1.01 (0.58–1.78)	0.969	0.99 (0.44–2.22)	0.975
3	1.03 (0.59–1.81)	0.910	0.73 (0.38–1.40)	0.343	1.09 (0.43–2.80)	0.855

Academic	0.55 (0.33–0.92)	0.022*	1.47 (0.86–2.52)	0.156	0.791 (0.34–1.82)	0.583
BI classification on admission						
85–100	reference		reference		reference	
0–55	1.55 (0.77–3.15)	0.221	1.60 (0.80–3.18)	0.181	1.32 (0.61–2.85)	0.474
60–80	1.45 (0.50–4.24)	0.496	0.65 (0.19–2.18)	0.487	1.20 (0.38–3.79)	0.761
Medical history						
Diabetes mellitus	1.19 (0.63–2.25)	0.592	0.81 (0.42–1.53)	0.510	1.30 (0.56–3.01)	0.544
Hypertension	1.03 (0.60–1.75)	0.919	1.06 (0.65–1.73)	0.813	1.12 (0.57–2.20)	0.749
Cerebral infarction	0.47 (0.06–3.75)	0.480	1.00		1.24 (0.25–6.14)	0.790
Angina pectoris	2.29 (0.64–8.24)	0.205	0.16 (0.02–1.25)	0.081	0.52 (0.11–2.48)	0.416
Chronic heart disease	N/A		1.96 (0.41–9.34)	0.400	N/A	
Internal oral medication on admission						
Antiplatelet	11.43 (5.79–22.56)	<0.001*	6.36 (3.49–11.61)	<0.001*	4.07 (1.74–9.54)	0.001*
Anticoagulation	4.06 (1.75–9.43)	0.001*	2.21 (0.99–4.97)	0.054	4.27 (1.86–9.82)	0.001*
Statin	0.54 (0.23–1.30)	0.170	1.09 (0.56–2.12)	0.804	0.88 (0.34–2.25)	0.783

Abbreviations: BI, Barthel index; BMI, body mass index; ICH, Intracerebral hemorrhage; IQR, interquartile range; No., number; SAH, subarachnoid hemorrhage. * p<0.05.
