# Supplementary material for: Surgical Risk in Elderly Patients with Meningiomas in Japan
Source: J Clin Med. 2024 May 14;13(10):2882. doi: 10.3390/jcm13102882 (PMC11122238; doi:10.3390/jcm13102882)
Supplement: Supplementary file 1 [file jcm-13-02882-s001.zip › Table S2.pdf]

Supplementary Table S2. Multivariate logistic regression analyses for BI deterioration based on age groups.

**Variable**	**BI deterioration**					
	**Nonelderly: < 65 (n=4378)**		**Pre-elderly: 65–74 (n=2421)**		**Elderly: ≥75 (n=1339)**	
	**OR (95% CI)**	**p**	**OR (95% CI)**	**p**	**OR (95% CI)**	**p**
Sex male	1.13 (0.87–1.46)	0.368	0.99 (0.75–1.30)	0.918	1.03 (0.77–1.37)	0.859
Age (year)	1.01 (1.00–1.02)	0.203	1.05 (1.00–1.10)	0.034*	1.08 (1.04–1.12)	<0.001*
BMI classification						
<18.5	1.00 (0.64–1.57)	0.983	1.20 (0.75–1.91)	0.451	1.27 (0.78–2.04)	0.335
18.5–24.9	reference		reference		reference	
25–29.9	1.34 (1.01–1.77)	0.041*	0.84 (0.61–1.14)	0.261	0.92 (0.67–1.28)	0.634
30≤	0.92 (0.55–1.55)	0.762	0.91 (0.49–1.67)	0.751	0.70 (0.31–1.56)	0.383
Location						
Convexity	reference		reference		reference	
Falx	2.16 (1.45–3.23)	<0.001*	1.47 (0.97–2.22)	0.071	0.96 (0.59–1.57)	0.869
Parasagittal	2.57 (1.81–3.65)	<0.001*	1.81 (1.24–2.65)	0.002*	1.37 (0.87–2.15)	0.173
Lateral	1.70 (1.11–2.59)	0.014*	1.47 (0.96–2.24)	0.075	0.98 (0.63–1.52)	0.916
Midline	1.37 (0.87–2.17)	0.173	1.35 (0.87–2.08)	0.178	0.85 (0.46–1.58)	0.616
Posterior fossa	1.35 (0.90–2.03)	0.148	1.16 (0.78–1.73)	0.468	1.35 (0.93–1.96)	0.117
Deep	3.12 (1.70–5.74)	<0.001*	2.94 (1.31–6.57)	0.009*	1.38 (0.40–4.73)	0.610
Hospital volume						
1	reference		reference		reference	
2	1.00 (0.73–1.37)	0.989	0.99 (0.73–1.35)	0.970	0.99 (0.72–1.37)	0.961
3	1.17 (0.83–1.65)	0.361	0.86 (0.61–1.22)	0.401	1.01 (0.69–1.47)	0.974

Academic	1.05 (0.79–1.39)	0.733	1.27 (0.95–1.71)	0.106	0.82 (0.59–1.15)	0.243
BI classification on admission						
85–100	reference		reference		reference	
0–55	1.41 (0.90–2.20)	0.135	1.13 (0.75–1.70)	0.550	0.47 (0.32–0.69)	<0.001*
60–80	3.19 (1.95–5.20)	<0.001*	2.74 (1.75–4.28)	<0.001*	1.82 (1.19–2.79)	0.006*
Medical history						
Diabetes mellitus	1.39 (0.95–2.04)	0.086	1.11 (0.80–1.55)	0.541	0.98 (0.67–1.42)	0.904
Hypertension	0.87 (0.63–1.21)	0.404	0.93 (0.71–1.22)	0.615	0.94 (0.71–1.24)	0.650
Cerebral infarction	1.59 (0.59–4.29)	0.362	1.89 (0.92–3.90)	0.084	1.37 (0.61–3.10)	0.445
Angina pectoris	0.69 (0.21–2.29)	0.548	0.75 (0.35–1.61)	0.456	1.37 (0.77–2.44)	0.283
Chronic heart disease	0.93 (0.20–4.22)	0.924	0.64 (0.18–2.33)	0.501	1.21 (0.55–2.68)	0.640
Internal oral medication on admission						
Antiplatelet	2.03 (1.05–3.93)	0.035*	1.35 (0.82–2.23)	0.241	1.08 (0.68–1.72)	0.751
Anticoagulation	2.55 (1.36–4.78)	0.003*	3.71 (2.39–5.76)	<0.001*	1.34 (0.82–2.17)	0.240
Statin	1.00 (0.61–1.63)	0.989	1.26 (0.87–1.82)	0.227	1.22 (0.82–1.81)	0.329

Abbreviations: BI, Barthel index; BMI, body mass index; ICH, Intracerebral hemorrhage; IQR, interquartile range; No., number; SAH, subarachnoid hemorrhage. * $p<0.05$.
